# Influencing factors of digital health technology anxiety in the elderly: a systematic review and meta-analysis

**DOI:** 10.3389/fpsyg.2025.1645753

**Published:** 2025-10-02

**Authors:** Ying Han, Jiquan Zhang, Wei Qing, Shouqi Zheng, Lin Xia, Yuxin Li, Lin He

**Affiliations:** ^1^School of Nursing, Chengdu University of Traditional Chinese Medicine, Chengdu, China; ^2^Nephrology Department, Deyang People's Hospital, Deyang, China; ^3^School of Nursing, North Sichuan Medical College, Nanchong, China; ^4^Department of Nursing, Deyang People's Hospital, Deyang, China

**Keywords:** elderly, digital health technology, technology anxiety, influencing factor, meta-analysis

## Abstract

**Background:**

With the acceleration of global population aging, the widespread application of digital health technologies provides new opportunities for health management of older adults. However, many older adults generally face “digital health technology anxiety,” which is characterized by irrational fear and resistance to emerging technologies, resulting in significantly lower rates of technology adoption than younger adults and increased health inequalities. The factors influencing digital health technology anxiety in the elderly were comprehensively evaluated through meta-analysis to provide an evidence-based foundation for developing targeted intervention measures.

**Methods:**

According to the Preferred Reporting Items for Systematic Reviews and Meta-Analyses (PRISMA) statement, the systematic search of Chinese and English databases (as of February 2025) included cross-sectional OR cohort studies of older adults ≥60 years old, and the outcome measures were factors influencing technology anxiety (reported as odds ratios [OR] with and corresponding 95% confidence intervals [CI]). Statistical analyses were performed using RevMan 5.3 software, combining effect sizes by fixed or random effects models. Heterogeneity was tested using the *I*^2^ test. In addition, sensitivity analyses and publication bias assessment were performed.

**Results:**

Following the screening process, 11 studies were included in the analysis. The meta-analysis showed that the following factors significantly associated with technology anxiety: age (OR = 1.09, 95%CI 1.03–1.14), digital health literacy (OR = 0.67, 95%CI 0.49–0.92), monthly income (OR = 0.73, 95%CI 0.62–0.87), household registration (OR = 0.19, 95%CI 0.08–0.45), family support (OR = 0.85, 95%CI 0.81–0.90), social network (OR = 0.60, 95%CI 0.54–0.66), information application ability (OR = 0.46, 95%CI 0.28–0.74), and self-efficacy (OR = 0.96, 95%CI 0.92–0.99). Sensitivity analyses showed consistent overall results, although there was some variation in the size of the age group.

**Discussion:**

Digital health technology anxiety among the elderly is influenced by multiple factors, including individual characteristics, technological capabilities, and social support. Designing for the elderly, low-income, and rural populations is essential to improve digital literacy, optimize age-appropriate designs, and strengthen family-community support, ultimately alleviating anxiety. Future studies need to expand the sample size and include longitudinal data to validate the causal association.

**Systematic review registration:**

The protocol for this systematic review has been registered in PROSPERO (CRD42025649793, available at: https://www.crd.york.ac.uk/PROSPERO/search).

## 1 Introduction

Population aging is accelerating globally. By 2050, it is projected that 1 in 6 people will be over the age of 65 (16%), compared to 1 in 11 (9%) in 2019 (United Nations, 2019). This demographic shift has already significantly increased the financial strain on the public health system. Moreover, older adults have a higher prevalence of multiple chronic diseases compared to younger populations (Meng et al., 2022). These demographic changes have not only led to health burdens and an increased demand for ongoing, long-term health management for the elderly but have also driven the development of digital health technologies (Huguet et al., 2023).

It is worth noting that aging is not only a demographic phenomenon but also involves multiple dimensions such as quality of life, emotional wellbeing, social participation, and physical health. Achieving active and healthy aging relies on non-pharmacological strategies, including social support, leisure activities, and physical exercise. These factors have been proven essential for improving life satisfaction and quality of life in older adults (Parra-Rizo et al., 2022; Sanchís-Soler et al., 2025). Therefore, while addressing health needs, it is also important to pay attention to social participation and daily activities. This further highlights the significance of exploring the use of digital health technologies to support healthy and active aging.

With the acceleration of the global population aging process and the rapid development of digital technology, providing technology-enabled nursing services for the elderly has become an inevitable trend to meet the health needs of the elderly (Luo et al., 2025). The COVID-19 pandemic dramatically accelerated the uptake of digital health technologies. For example, telehealth use among adults aged 70+ increased from 4.6% pre-pandemic to 21.1% during the pandemic (Choi et al., 2022). Digital health technologies refer to innovative forms of health-related services delivered through information and communication technologies (ICT), including mobile health applications, telemedicine platforms, electronic health record systems, artificial intelligence-assisted diagnostic tools, smart wearable devices, medication management systems, and smart home monitoring devices (Kim et al., 2023). These technologies not only support daily health management for the elderly but also promote early disease screening and the widespread adoption of telemedicine services (Park et al., 2025), thereby enhancing independence, safety, and overall quality of life for the elderly (Kang et al., 2021). Additionally, they can improve time flexibility and reduce costs compared to traditional medical treatments (Abernethy et al., 2022). This emerging industry, which applies digital transformation to the medical field, has attracted international attention (Wang and Luan, 2022). It offers safe and affordable medical services for the elderly, providing them with great convenience (Klaver et al., 2021). For example, mobile health programs can help elderly people easily schedule medical appointments and access their health records (Kruse et al., 2017).

Despite the potential benefits of digital health technologies, older adults face significant barriers to adapting to these emerging technologies. One of the most important barriers to the adoption and use of digital health technologies is ‘digital health technology anxiety,’ which refers to an individual’s irrational fear of or anxiety about new technologies and resistance to technological stimuli that change existing behaviors (Khasawneh, 2018). This is manifested in the fear of the complexity involved in operating the equipment, concerns about privacy breaches, and resistance to technology as a substitute for traditional healthcare services. In the elderly population, this manifests as nervousness and hesitancy during the actual use, leading to negative experiences and reduced self-confidence (Tsai et al., 2020). Studies have shown that digital health technology usage remains significantly lower among older age groups compared to younger age groups (Hauk et al., 2018). International studies have shown extremely high rates of digital exclusion among older persons globally, ranging from 23.8% in Denmark to 96.9% in China. It suggests that a significant proportion of older adults do not have access to digital technologies or services, which in turn may widen inequalities in the use of health services (Lu et al., 2022). In addition, these inequalities can exacerbate social disparities among older people and contribute to their social exclusion, underscoring the urgency of public policy interventions to promote the equitable adoption of digital health technologies by older persons (Yang et al., 2024).

In recent years, researchers have tended to focus more on attitudes and willingness to use digital health technologies among the elderly. Empirical studies on the technology anxiety of the elderly are still insufficient. Some studies have explored the influencing factors of digital health technology anxiety in the elderly, including individual characteristics (such as age, education level, and gender), technical attributes (such as ease of use and functionality), and social environment (such as family support and social resources) (Felber et al., 2024). However, the majority of existing studies are cross-sectional, resulting in fragmented and inconsistent findings. Differences in study design, measurement tools, and populations make it difficult to determine which factors are most critical in influencing technology anxiety among older adults.

To address this issue, this study quantitatively integrates the established literature through meta-analysis to more precisely identify the primary risk factors and their effect sizes, assess the stability of the results, and reveal potential heterogeneity. This approach not only helps to make up for the limited conclusions of single studies but also provides an evidence-based foundation for future interventions and public policy formulation, especially in the areas of digital literacy enhancement, aging-friendly technology design, and social support reinforcement for high-risk populations.

## 2 Materials and methods

This meta-analysis was conducted in accordance with the recommendations of the PRISMA guidelines and is registered on the International Prospective Register of Systematic Reviews (PROSPERO) website (Registration number: CRD42025649793).

### 2.1 Literature retrieval strategy

**Figure 1:**
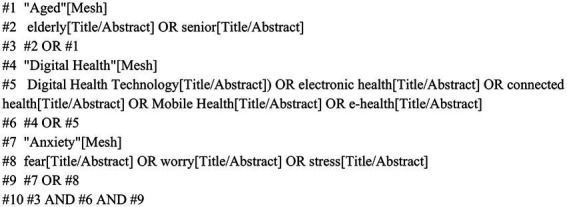
PubMed search strategy.

Studies were obtained by searching the online databases such as China National Knowledge Infrastructure (CNKI), Wanfang Data Knowledge Service Platform, Vertically Integrated Projects (VIP) database, China Biomedical Literature Database, PubMed, Web of Science, Embase, and Cochrane. The search period was from the establishment of the database to February 2025. The search was conducted by combining subject words and free words and adjusting them according to the characteristics of each database. The search strategy was as follows: (((“Aged”[Mesh]) OR ((elderly[Title/Abstract]) OR (senior[Title/Abstract]))) AND ((“Digital Health”[Mesh]) OR (((((Digital Health Technology[Title/Abstract]) OR (electronic health[Title/Abstract])) OR (connected health[Title/Abstract])) OR (Mobile Health[Title/Abstract])) OR (e-health[Title/Abstract])))) AND ((“Anxiety”[Mesh]) OR (((fear[Title/Abstract]) OR (worry[Title/Abstract])) OR (stress[Title/Abstract]))). The PubMed retrieval diagram is shown in Figure 1.

### 2.2 Inclusion and exclusion criteria

The inclusion criteria were as follows: ① Study type: cross-sectional studies and, cohort studies; ② Study subjects: individuals aged 60 years and older; ③ Research theme: Influencing factors or risk factors of digital health technology anxiety in the elderly; ④ Outcome indicators: The literature must provide extractable effect size data, OR values, and 95%CI or original data that can be converted into the aforementioned metrics.

The exclusion criteria were as follows: ① non-Chinese or English literature; ② the original data that were incomplete or could not be extracted; ③ no technical anxiety scale was used, or no tools were explicitly reported for assessing reliability and validity.

### 2.3 Literature screening and data extraction

Two researchers independently screened the literature, extracted the data and cross-checked their findings. Differences, if any, were resolved through discussion or mediation by a third party. Data such as the author name, publication year, study type, region, sample size, influencing factors, and outcome indicators were extracted.

### 2.4 Quality assessment of literature

The Newcastle-Ottawa Scale (NOS) was used to evaluate the quality of the cohort studies. The scale was divided into three parts and eight items, with a total score of 9 points. Scores ranging from 0–3 were classified as low quality, 4–6 as medium quality, and 7–9 as high quality (Stang, 2010). The quality of the included cross-sectional studies was evaluated using the 11-item methodological checklist developed by the U.S. Agency for Healthcare Research and Quality (AHRQ). A total of 11 items were included in the AHRQ survey, with a total score of 11. Scores ranging from 0–3 were classified as low quality, 4–7 as medium quality, and 8–11 as high quality (Zeng et al., 2012).

### 2.5 Statistical analysis

Data were analyzed using RevMan 5.3 software. The effect sizes used were the odds ratios (ORs) and 95% CIs of the factors affecting digital health technology anxiety in older adults. Some original studies reported the *β* coefficient of logistic regression. We obtained the OR value using the transformation formula suggested by Bland and Altman (2000). *I^2^* was used to determine the magnitude of the heterogeneity. When *I*^2^ was ≥ 50% and *p* < 0.1, heterogeneity was observed in the literature, which was analyzed using a random-effects model for the combined analysis. When *I*^2^ was < 50% and *p* > 0.1, there was less heterogeneity in the literature, which was analyzed using a fixed-effects model for the combined analysis. The exclusion of the literature was done by removing the studies individually for the sensitivity analysis. A *p*-value of < 0.05 indicated statistical significance. Funnel plots combined with the Egger regression test were used to assess publication bias. The criterion for significant publication bias was set at *p* < 0.05.

## 3 Results

### 3.1 Study selection process

**Figure 2:**
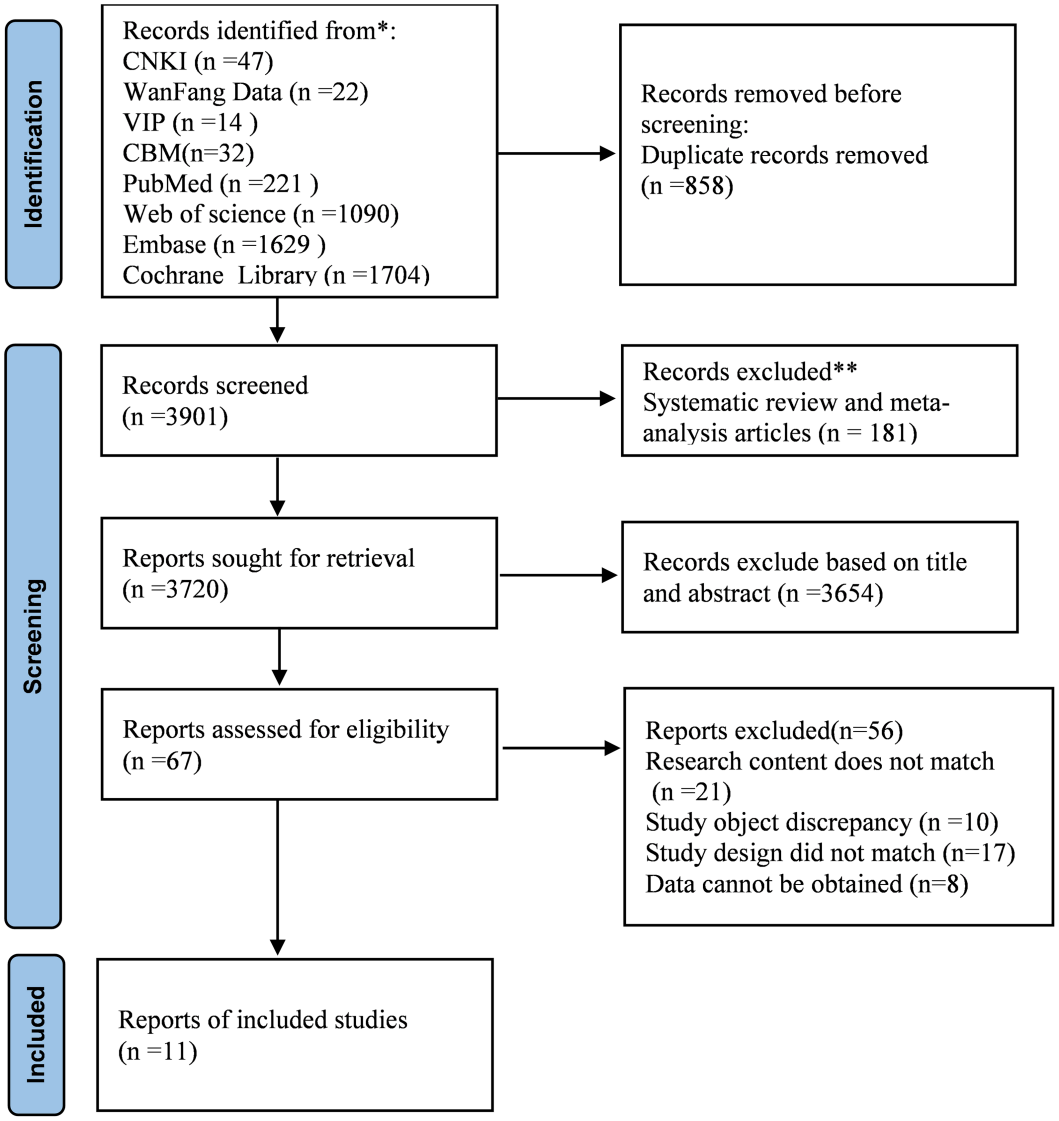
PRISMA flow diagram of the literature search and study selection process.

Figure 2 shows the process of literature screening and the reasons for excluding them. A total of 4,759 citations were included in this study. After eliminating duplicate entries, 3,901 records were entered during the initial screening stage. After checking the titles and abstracts of each paper and eliminating inconsistent literature, 67 studies were found to be related to the research topic. Among them, 21 studies were excluded because their contents did not involve anxiety in digital health technologies, 10 studies had subjects who were not elderly, 17 studies did not match the types, and 8 studies lacked original data. Finally, the meta-analysis included 11 original studies.

### 3.2 Basic characteristics and quality evaluation of the included studies

A total of 11 studies were included—10 cross-sectional studies and 1 cohort study—published between 2018 and 2024, with a combined sample size of 4,868 cases. The countries included in the study were China, Israel, and Sweden. The quality of these 11 studies was assessed, and the scores ranged from 4 to 8, with 3 studies having a high-quality rating and 8 studies with a medium-quality rating. The outcomes of the 11 included studies were measured using the Technophobia Scale developed by Khasawneh (2018) or Spagnolli et al. (2014). The basic characteristics and quality assessment of the literature are presented in Table 1.

**Table 1:** Basic characteristics and quality assessment of the included literature (*n* = 11).

Inclusion of literature	Year of publication	Type of study	Sample size	Country	Influencing factors	Quality of literature
Xie et al. (2023)	2023	Cross-sectional study	212	China	①②③	Medium
Li et al. (2023)	2023	Cross-sectional study	160	China	④⑤⑥⑦	Medium
Wu et al. (2023)	2023	Cross-sectional study	318	China	②⑥⑧⑨⑪	Medium
Chen et al. (2024)	2024	Cross-sectional study	1,222	China	③④⑧⑨⑫⑬	High
Wang et al. (2024)	2024	Cross-sectional study	257	China	②⑧⑩⑭	Medium
Peng et al. (2023)	2023	Cross-sectional study	606	China	①②③④⑤⑥⑦⑩	High
Guo et al. (2024)	2024	Cross-sectional study	320	China	①③⑤⑦⑮	Medium
Tang et al. (2023)	2023	Cross-sectional study	291	China	①③④⑥⑯	Medium
Sun et al. (2024)	2024	Cross-sectional study	552	China	⑰	Medium
Nimrod (2018)	2018	Cross-sectional study	537	Israel	②⑱	Medium
Berner et al. (2023)	2023	Cohort study	393	Sweden	⑲	High

① Age; ② Education level; ③ Digital health literacy; ④ Monthly income; ⑤ Household registration; ⑥ Family support; ⑦ Social networks; ⑧ Ability to acquire and evaluate information; ⑨ Information application ability; ⑩ Self-efficacy; ⑪ Gender; ⑫ Reserves of knowledge; ⑬ Reservoir of resources; ⑭ Occupation; ⑮ The active aging level; ⑯ Marital status; ⑰ Perceived usefulness; ⑱ Self-rated health status; and ⑲ Neurotic.

### 3.3 Meta-analysis results

Table 1 summarizes the 25 potential influencing factors involved in the included studies. However, the meta-analysis of this study only focused on 10 factors that were supported by a certain number of similar studies and met the conditions for quantitative synthesis: age, monthly income, household registration, social network use, and self-efficacy. These factors had little heterogeneity (*I*^2^ < 50%), and the fixed-effects model was used for the analysis. Digital health literacy, family support, and information application ability showed great heterogeneity (*I*^2^ ≥ 50%), and the random-effects model was selected for analysis. The results showed no statistical significance in educational level, information acquisition, and assessment ability (*p* > 0.05). Age, digital health literacy, monthly income, household registration, family support, social network use, information application ability, and self-efficacy were influencing factors of digital health technology anxiety in the elderly, and the combined OR values and 95%CI were statistically significant (*p* < 0.05). Details are presented in Table 2.

**Table 2:** Meta-analysis of factors associated with digital health technology anxiety in older adults.

Risk factor	Number of documents	Heterogeneity test		Effects model	The combined OR		Combined effect size test	
		*p*	*I^2^* (%)		OR	95%CI	*Z*	*p*
Age	5	0.20	34	Fixed	1.09	1.03 ~ 1.14	2.32	<0.01
Digital health literacy	4	0.10	51	Random	0.67	0.49 ~ 0.92	2.50	0.01
Monthly income	4	0.96	0	Fixed	0.73	0.62 ~ 0.87	3.69	<0.01
Household registration	3	0.26	22	Fixed	0.19	0.08 ~ 0.45	3.74	<0.01
Family support	4	0.006	76	Random	0.85	0.81 ~ 0.90	5.88	<0.01
Social networks	7	0.49	0	Fixed	0.60	0.54 ~ 0.66	10.44	<0.01
Ability to acquire and evaluate information	2	0.07	70	Random	0.46	0.28 ~ 0.74	3.17	<0.01
Self-efficacy	2	0.32	0	fixed	0.96	0.92 ~ 0.99	2.28	0.02

The results include the number of studies, heterogeneity statistics (I^2^ and *p*-value), effect model, pooled odds ratio with 95% confidence interval, and significance test.

### 3.4 Sensitivity analysis

#### 3.4.1 Leave-one-out analysis

We conducted a sensitivity analysis using a stepwise exclusion method for studies with *I^2^* ≥ 50%. The heterogeneity of the studies (Guo et al., 2024) excluded from digital health literacy decreased, and *I*^2^ changed from 51 to 0. When (Wu et al., 2023) it was excluded from family support, the heterogeneity of the study decreased, and *I*^2^ changed from 76 to 0. These results indicate that the removal of literature may have been the source of heterogeneity after combining the data (Table 3).

**Table 3:** The old digital health technology anxiety sensitivity analysis of influencing factors.

Risk factor	Eliminate	References were included after exclusion	Before excluding				After exclusion				*P*
			Effect model	OR (95%CI)	Heterogeneity		Effect model	OR (95%CI)	Heterogeneity		
					*p*	*I^2^* (%)			*p*	*I^2^* (%)	
Digital health literacy	Guo et al. (2024)	3	Random	0.67 (0.49–0.92)	0.10	51	Fixed	0.75 (0.64–0.88)	0.78	0	<0.01
Family support	Wu et al. (2023)	3	Random	0.85 (0.81–0.90)	0.006	76	Fixed	0.85 (0.81–0.90)	0.92	0	<0.01

#### 3.4.2 Transformative statistical models

After removing literature individually, a sensitivity analysis of the relevant factors extracted in all studies was carried out by converting the random- and fixed-effects models. The consistency of other factors, except age, was good, which indicated that the results of this study were dependable (Table 4).

**Table 4:** Sensitivity analysis of meta-analysis results.

Risk factor	Fixed-effect model			Random effects model			Stability
	OR	95%CI	*p*	OR	95%CI	*p*	
Age	1.09	1.03–1.14	<0.01	1.08	0.92–1.28	0.33	Instability
Digital health literacy	0.75	0.64–0.88	<0.01	0.75	0.64–0.88	<0.01	Stabilize
Monthly income	0.73	0.62–0.87	<0.01	0.73	0.62–0.87	<0.01	Stabilize
Household registration	0.19	0.08–0.45	<0.01	0.22	0.06–0.81	0.02	Stabilize
Family support	0.85	0.64–0.88	<0.01	0.85	0.81–0.90	<0.01	Stabilize
Social networks	0.60	0.54–0.66	<0.01	0.60	0.51–0.66	<0.01	Stabilize
Ability to acquire and evaluate information	0.48	0.38–0.62	<0.01	0.46	0.28–0.74	<0.01	Stabilize
Self-efficacy	0.96	0.92–0.99	0.02	0.96	0.92–0.99	0.02	Stabilize

### 3.5 Publication bias assessment

**Figure 3:**
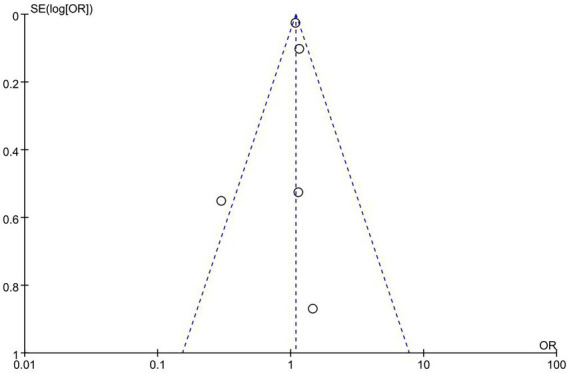
Funnel plot for the assessment of publication bias for the factor ‘age.’ The plot visualizes the standard error of the log (OR) against the odds ratio (OR) of individual studies. The symmetrical distribution of the points suggests no significant publication bias.

In this study, funnel plot analysis was performed based on age, and the results showed a roughly symmetrical distribution of points, with an Egger regression test of *p* > 0.05, suggesting that there was no significant publication bias. However, the small number of studies included in this meta-analysis (<10) may limit the validity of the test. The funnel plot is shown in Figure 3.

## 4 Discussion

In this meta-analysis, 11 studies were integrated to explore the factors influencing digital health technology anxiety among older adults, and 8 significant correlations were identified. It covers various factors, including demographic characteristics (age, income, domicile, and self-efficacy), technological attributes (digital health literacy and information application skills), and socioenvironmental factors (family support, and social networks). These findings suggest that the development of digital health technology anxiety among older adults is the result of a multifactorial approach. This anxiety is not only influenced by individual characteristics but is also closely related to the technology itself and the surrounding social environment.

### 4.1 The effect of general demographic information on technology anxiety

The results of this study show that increasing age is a risk factor for technology anxiety, which is consistent with previous studies (Sun and Ye, 2024). Older adults have more difficulty adapting to new technologies due to their declining cognitive function and reduced learning ability. Other studies have shown insignificant age differences, which may be related to differences in the sample stratification, education level, prior experience with technology exposure, or family support status. Elderly people (over 80 years old) may be less accepting of technological change and experience more pronounced technology anxiety due to significant cognitive decline (Choi and Dinitto, 2013). Educational attainment moderates digital health technology anxiety among older adults of different ages. Older adults with higher levels of literacy have an advantage in adopting health technologies, whereas those with lower levels of education limit their use (Bertolazzi et al., 2024). Therefore, future research should consider multifactorial interactions to clarify the actual role of age in different contexts using subgroup analyses or moderation models. A longitudinal design was used to observe the adaptation process of older adults of different ages and to identify key turning points. This study provides a precise basis for developing digital health interventions for older adults.

Elderly people with low income and rural household registration are at a higher risk of experiencing anxiety, which also reflects the limitation of technological accessibility due to economic resources and regional differences. Older adults with lower income levels lack the financial means to purchase smart devices or pay for digital services (Choi and Dinitto, 2013). This financial barrier limits their access to digital health technologies and indirectly increases their anxiety. Older people with rural household registration are at a disadvantage in terms of accessibility due to differences in digital infrastructure between urban and rural areas, making them susceptible to a sense of digital deprivation (Peng et al., 2023). The influence of household registration is rarely presented in the previous literature, which may be a unique urban–rural disparity phenomenon in China. More localized studies are needed to further investigate and validate this phenomenon.

Self-efficacy influences older adults’ judgments regarding their ability to successfully use digital health technologies (Korkmaz Aslan et al., 2021). This concept aligns with social cognitive theory. Self-efficacy shapes individuals’ perceptions of task difficulty and their emotional responses. Older adults with low self-efficacy believe that they lack the ability to operate digital devices. They often feel anxious regarding the use of new technologies. This anxiety reduces their willingness to adopt digital tools (Askari et al., 2020). In contrast, older adults with high self-efficacy view new technology as a challenge that they can overcome (Pan et al., 2016). They feel more motivated to learn and try. They are also more likely to persist even when they face difficulties.

In the future, more attention should be given to older people from rural households who have low income and low self-efficacy. Inclusive digital health services should be promoted through the provision of government subsidies for the cost of smart devices or Internet access. Age-appropriate interfaces should be designed to reduce operational complexity, and the progress of older individuals’ technological learning should be recorded. Their achievements should be praised in a timely manner to increase their sense of participation.

### 4.2 The effect of technology attributes on technology anxiety

Digital health literacy refers to “the ability to search, find, understand, and evaluate health information from electronic resources, and apply the acquired knowledge to deal with or solve health problems” (Norman and Skinner, 2006). Previous studies have shown that the overall health literacy of the elderly is low (Liu et al., 2022). This lack of literacy has hindered the development of digital health technology and caused technological anxiety among the elderly. This is consistent with Luo’s “Literacy–Efficacy” mediation model (Luo et al., 2025). Older adults with high digital health literacy can better understand and use digital technologies to manage their health. They have more confidence when facing technology and naturally feel less anxious. Digital health literacy is often measured using the eHealth Literacy Scale (eHEALS), which has been widely applied to older populations. Our study shows that higher literacy levels are linked to greater confidence in using technology and lower anxiety levels. However, barriers such as cognitive decline, limited educational background, financial burden, privacy concerns, and complex interfaces continue to hinder the development of digital health literacy in older adults (Bertolazzi et al., 2024; Çöme et al., 2025). Strategies to address this may include community-based digital skills training, intergenerational family support, digital guidance from healthcare institutions, and policy initiatives that promote an age-friendly design.

Information application ability is highly correlated with digital health literacy, and older adults with high digital health literacy have stronger information application abilities. Older adults with good information application abilities can more efficiently utilize digital health technology to meet their own health needs and encounter fewer difficulties in the process of using it, which reduces their anxiety.

The Technology Acceptance Model (TAM) points out that (Ammenwerth, 2019) an individual’s “perceived usefulness” and “perceived ease of use” of technology are the core factors determining technology adoption. Digital health literacy and information application ability are indicators of perceived usefulness; the higher these levels, the better the elderly can adapt to digital health technology and reduce technology anxiety. This suggests that improving the digital health literacy of older adults is a key way to alleviate anxiety and that targeted training programs, such as community-based digital health knowledge lectures and online learning platforms, can help older adults improve their literacy (Czaja et al., 2013).

### 4.3 Influence of the social environment on technological anxiety

Inadequate family support and a lack of social networks significantly increased anxiety, validating the social support theory that assistance from family members or peers can increase older adults’ confidence in using technology (Lorig et al., 1999). Family support comes from three aspects: financial support, life support, and psychological support (Xu, 2019). When older adults learn to use digital health technology, family members help them adapt through encouragement, patient communication, and hands-on assistance. The more the family members can alleviate the stress associated with adapting to the technology, the more significantly their level of technological anxiety is reduced (Peng et al., 2023).

Findings show that the higher the frequency of social network use among older adults, the lower is their level of technology anxiety (Li et al., 2023). A higher frequency of network use reduces older adults’ fear of digital health technology and increases their trust, leading to improved physical and mental health (Zhao and Liu, 2020). This suggests that we can actively mobilize the social relationship network of the elderly, guide their family members, relatives, and friends to participate, and provide digital feedback for the elderly to help them better understand and adapt to digital health technology and improve the enthusiasm of the elderly to use it (Portz et al., 2019). Specific practical strategies can be implemented. For example, family-based programs can be established to involve children or other family members in the elderly’s digital health learning, providing operational guidance and psychological support; community support groups can be formed to facilitate peer-to-peer exchange of usage experiences and sharing of learning resources among the elderly; and community activities can be organized to offer hands-on practice and experiential opportunities, helping the elderly gradually become familiar with digital health tools. It is important to note that the effectiveness of social support may vary due to cultural and regional differences such as differences in family structure, intergenerational relationships, and access to community resources, which may limit the generalizability of the research findings. Therefore, future research should focus on the influence of socioenvironmental factors across different social contexts.

### 4.4 Sensitivity analysis

The results of the sensitivity analysis showed that the heterogeneity of digital health literacy and family support was greatly affected by some studies, and heterogeneity was significantly reduced after the removal of relevant literature. Except for age, the stability of the other factors was relatively good under the transformation of different effect models, indicating that the results of this study were generally reliable. The stability of the age factor was poor under different models, which may be related to the age group differences in the study. The high heterogeneity observed for some variables may stem from differences in measurement tools or scoring methods, variations in sample characteristics (e.g., age, income, and urban/rural residence), and cultural factors that affect social support and digital health literacy. This level of heterogeneity further emphasizes the importance of maintaining methodological consistency in future studies. We recommend that future studies adopt standardized research protocols, use validated measurement tools consistently, and report study characteristics more transparently to reduce differences and improve the quality of evidence.

The findings of this study align with the Technology Acceptance Model (TAM). High levels of digital health literacy and information application skills can enhance older adults’ perceived usefulness and perceived ease of use of technology, thereby reducing anxiety and promoting technology adoption. Additionally, self-efficacy was found to play a significant role in this study. This finding aligns with the emphasis on autonomy and a sense of control in the self-management model. This suggests that older adults who maintain self-efficacy during technology use are more likely to overcome their anxiety and improve their adaptability. Combining the TAM with the self-management framework may offer a more comprehensive perspective on understanding the mechanisms underlying older adults’ anxiety toward digital health technologies.

Overall, this study systematically analyzed the multidimensional factors influencing older adults’ anxiety toward digital health technologies, including demographic characteristics, technological attributes, and socioenvironmental factors. By integrating research findings with theoretical models, this study contributes to the development of theories while offering guidance for practical interventions.

## 5 Research limitations

The number of studies included in this study is limited, which reflects the current evidence base in this emerging field. Existing studies are scattered with heterogeneous designs and occasionally inconsistent findings, making it difficult to draw generalizable conclusions from individual studies. By pooling data from 4,868 participants, our meta-analysis provides greater statistical power than single studies and highlights consistent factors associated with digital health technology anxiety among older adults. Moreover, sensitivity analyses and publication bias assessments supported the robustness of the majority of results. Some factors (such as information application ability) were supported by only two studies, and the statistical efficacy was insufficient. It is worth noting that the majority of the included studies were cross-sectional, which limited the inference of causal relationships and could only suggest correlations between variables. It should also be noted that the majority of the studies included in this review originated from China, which, to some extent, limits the global generalizability of the findings. This is because older adults’ acceptance of and anxiety about digital health technologies may be influenced by cultural contexts, healthcare systems, and technological usage environments. Consequently, the applicability of these results to other countries or cultural settings warrants cautious interpretation. Future studies should adopt more longitudinal designs or controlled trials to further verify causal pathways and mechanisms of action. Focusing solely on quantitative studies may limit conceptual richness, and qualitative studies could offer deeper insights into the lived experiences of older adults. Therefore, future research may benefit from a mixed-methods review that integrates both quantitative outcomes and qualitative narratives.

## 6 Conclusion

This study systematically analyzed the factors influencing digital health technology anxiety among older adults, confirming the key roles of age, digital health literacy, monthly income, household registration, family support, social network use, information application ability, and self-efficacy. Additionally, it outlines the direction for practical and feasible intervention strategies at multiple levels, including the government, communities, and medical institutions. The findings hold significant theoretical value and offer broad application prospects. At the specific role level, healthcare professionals can integrate digital health training into their daily health education and nursing practices; technology designers should consider age-friendly interface design and simplified operations; and policymakers can support the use of digital health tools by older adults through subsidies, resource allocation, and community projects. By focusing on these practical strategies and their feasibility, interventions can be made more effective, sustainable, and broadly applicable. Subsequent studies should focus on the dynamic effects of longitudinal data and technology iteration on digital health technology anxiety and further validation of the generalizability of the influencing factors across different social contexts.

## Data Availability

The original contributions presented in the study are included in the article/supplementary material, further inquiries can be directed to the corresponding author.
